# From Conception to Birth: Bridging Embryonic Development and Disease

**DOI:** 10.3390/medicina62040733

**Published:** 2026-04-12

**Authors:** Paschalis Theotokis, Soultana Meditskou, Maria Eleni Manthou

**Affiliations:** Department of Histology-Embryology, School of Medicine, Aristotle University of Thessaloniki, 54124 Thessaloniki, Greece; ptheotokis@auth.gr (P.T.); sefthym@auth.gr (S.M.)

Embryonic development represents a highly coordinated biological continuum, beginning at fertilization and extending through fetal life to birth. This process, governed by tightly regulated molecular and cellular mechanisms, underpins normal human development while simultaneously defining the origins of a wide spectrum of pathological conditions. This Special Issue, “From Conception to Birth: Embryonic Development and Disease”, brings together contributions that collectively explore this continuum, emphasizing the inseparable link between developmental biology and disease.

A foundational theme emerging from this collection is the molecular regulation of fertilization and early embryogenesis. The role of sperm-specific factors, such as phospholipase Cζ, highlights the finely tuned biochemical signaling required for oocyte activation and successful embryonic initiation [1]. Disruptions at this earliest stage, including chromosomal abnormalities and molecular defects, are further explored in the context of male infertility, where genetic, structural, and environmental contributors converge to impair reproductive potential [2]. Together, these studies underscore that the origins of developmental success (or failure) are established at, or even prior to, conception.

Extending beyond fertilization, this Special Issue addresses the increasingly complex landscape of assisted reproduction and embryo manipulation. Advances in assisted reproductive technologies (ART) have significantly improved reproductive outcomes; however, questions remain regarding the long-term safety of embryo culture conditions and cryopreservation techniques. The potential association between in vitro embryo handling and the risk of congenital anomalies reflects a critical interface between technological progress and developmental vulnerability [3], emphasizing the need for continued evaluation of both biological and procedural variables.

Another key dimension of this collection is the intersection between maternal health, pregnancy conditions, and developmental outcomes. High-risk pregnancies are examined as a major determinant of neonatal morbidity and mortality, highlighting how intrauterine environments shape developmental trajectories long before birth [4]. In parallel, fertility preservation strategies in female cancer patients illustrate the growing importance of safeguarding reproductive potential while navigating complex therapeutic interventions [5]. These contributions collectively reinforce the concept that embryonic and fetal development cannot be viewed in isolation but must be understood within the broader physiological and clinical context of the mother.

Importantly, this Special Issue also captures the transition from developmental biology to clinical intervention. Advances in fetal surgery demonstrate how early identification of congenital anomalies can be coupled with in utero therapeutic strategies, effectively redefining the boundaries between diagnosis and treatment [6]. Similarly, the investigation of prematurity through hemostatic profiling provides insight into the physiological adaptations and vulnerabilities of the developing organism, linking developmental immaturity with clinically significant outcomes in neonatal life [7].

Taken together, the contributions in this Special Issue illustrate that embryonic development is not a discrete biological phase but a continuum that extends from molecular events at fertilization to complex clinical outcomes in the neonatal period. They highlight how perturbations arising from genetic, environmental, technological, or physiological factors across different developmental stages can converge to influence developmental trajectories and disease susceptibility.

Looking forward, future research should aim to further integrate developmental biology with precision medicine approaches, fostering interdisciplinary collaboration between basic scientists, clinicians, and bioengineers. Such integration holds the potential to enable earlier diagnosis, improved risk stratification, and the development of targeted preventive and therapeutic strategies that begin even before birth, ultimately reshaping the clinical management of developmental disorders.

## Abbreviations

The following abbreviations are used in this manuscript:

ART	Assisted Reproductive Technologies
